# Ecological Shifts in Mediterranean Coralligenous Assemblages Related to Gorgonian Forest Loss

**DOI:** 10.1371/journal.pone.0102782

**Published:** 2014-07-23

**Authors:** Massimo Ponti, Rossella Angela Perlini, Vincenzo Ventra, Daniele Grech, Marco Abbiati, Carlo Cerrano

**Affiliations:** 1 Dipartimento di Scienze Biologiche, Geologiche e Ambientali, University of Bologna, UO CoNISMa, Ravenna, Italy; 2 Dipartimento di Scienze della Vita e dell’Ambiente, Polytechnic University of Marche, UO CoNISMa, Ancona, Italy; 3 ISMAR, Consiglio Nazionale delle Ricerche - Istituto di Scienze Marine, Bologna, Italy; Leibniz Center for Tropical Marine Ecology, Germany

## Abstract

Mediterranean gorgonian forests are threatened by several human activities and are affected by climatic anomalies that have led to mass mortality events in recent decades. The ecological role of these habitats and the possible consequence of their loss are poorly understood. Effects of gorgonians on the recruitment of epibenthic organisms were investigated by manipulating presence of gorgonians on experimental panels at 24 m depth, for *Eunicella cavolinii*, and at 40 m depth, for *Paramuricea clavata*, at two sites: Tavolara Island (Tyrrhenian Sea) and Portofino Promontory (Ligurian Sea). After 4 months, the most abundant taxa on the panels were encrusting green algae, erect red algae and crustose coralline algae at 24 m depth and encrusting brown algae and erect red algae at 40 m depth. Assemblages on the panels were significantly affected by the presence of the gorgonians, although effects varied across sites and between gorgonian species. Species diversity and evenness were lower on panels with gorgonian branches. Growth of erect algae and recruitment of serpulid polychaetes were also affected by the presence of the gorgonians, primarily at Tavolara. Crustose coralline algae and erect sponges were more abundant on *E. cavolinii* panels at 24 m depth, while encrusting bryozoans were more abundant on *P. clavata* panels at 40 m depth. Effects of gorgonians on recruited assemblages could be due to microscale modification of hydrodynamics and sediment deposition rate, or by a shading effect reducing light intensity. Gorgonians may also intercept settling propagules, compete for food with the filter-feeders and/or for space by producing allelochemicals. Presence of gorgonians mainly limits the growth of erect algae and enhances the abundance of encrusting algae and sessile invertebrates. Therefore, the gorgonian disappearances may cause a shift from assemblages characterised by crustose coralline algae to filamentous algae assemblages, decreasing complexity and resilience of coralligenous bioconstructions.

## Introduction

The coralligenous habitats are among the most typical ones in the Mediterranean Sea. They are bioconstructions, which host the highest level of benthic species diversity in the Mediterranean Sea [1], and Porifera is the richest taxon [2]. Coralligenous habitat structure has been clearly described by Riedl [3], who outlined a microscale zonation: an infaunal layer (boring and cryptic species) and an epibenthic layer with two main levels of complexity, one made by small encrusting organisms and a second made by species with vertical growth. Among the latter, several gorgonian species and sponges belonging to the genus *Axinella* mainly contributed to the building of the 3D complexity. When the density of these tree-like shaped organisms is high, environmental conditions (light, currents, sediment deposition rates, etc.) may be strongly altered, creating micro habitats that act as a shelter for many necto-benthic organisms [4], [5].

Integrity of large gorgonian forests can be affected by both natural disturbances (e.g. climatic anomalies [6], ocean acidification [7], exceptional storms [8]) and human activities (e.g. anchoring, SCUBA diving, recreational and commercial fishing [9], [10]). In the gorgonians, tissue injuries and physiological stress may increase the susceptibility to pathogens and facilitate settlements of epibionts [11], [12]. Over recent decades, these phenomena have led to gorgonian mass mortality events in the north-western Mediterranean Sea [13], [14], [15], [16]. Although these phenomena have been widely investigated, little is known about the consequences of gorgonian loss on the composition and abundance of other members of the coralligenous assemblages [17], [18], [19].

Predicting the consequences of loss of habitat forming species is critically important. Specifically, given the present threats to biological diversity such as habitat fragmentation, overharvesting and climate change.

With this aim, a field experiment was designed to investigate the effect of gorgonian forests on the settlement and early recruitment of epibenthic organisms on coralligenous habitats. Density of the two most common Mediterranean gorgonian octocorals, *Eunicella cavolinii* (Koch, 1887) and *Paramuricea clavata* (Risso, 1826), was experimentally manipulated in two locations, to quantify specific changes associated with their loss.

## Materials and Methods

### Experimental Set-up and Study Sites

**Figure 1:**
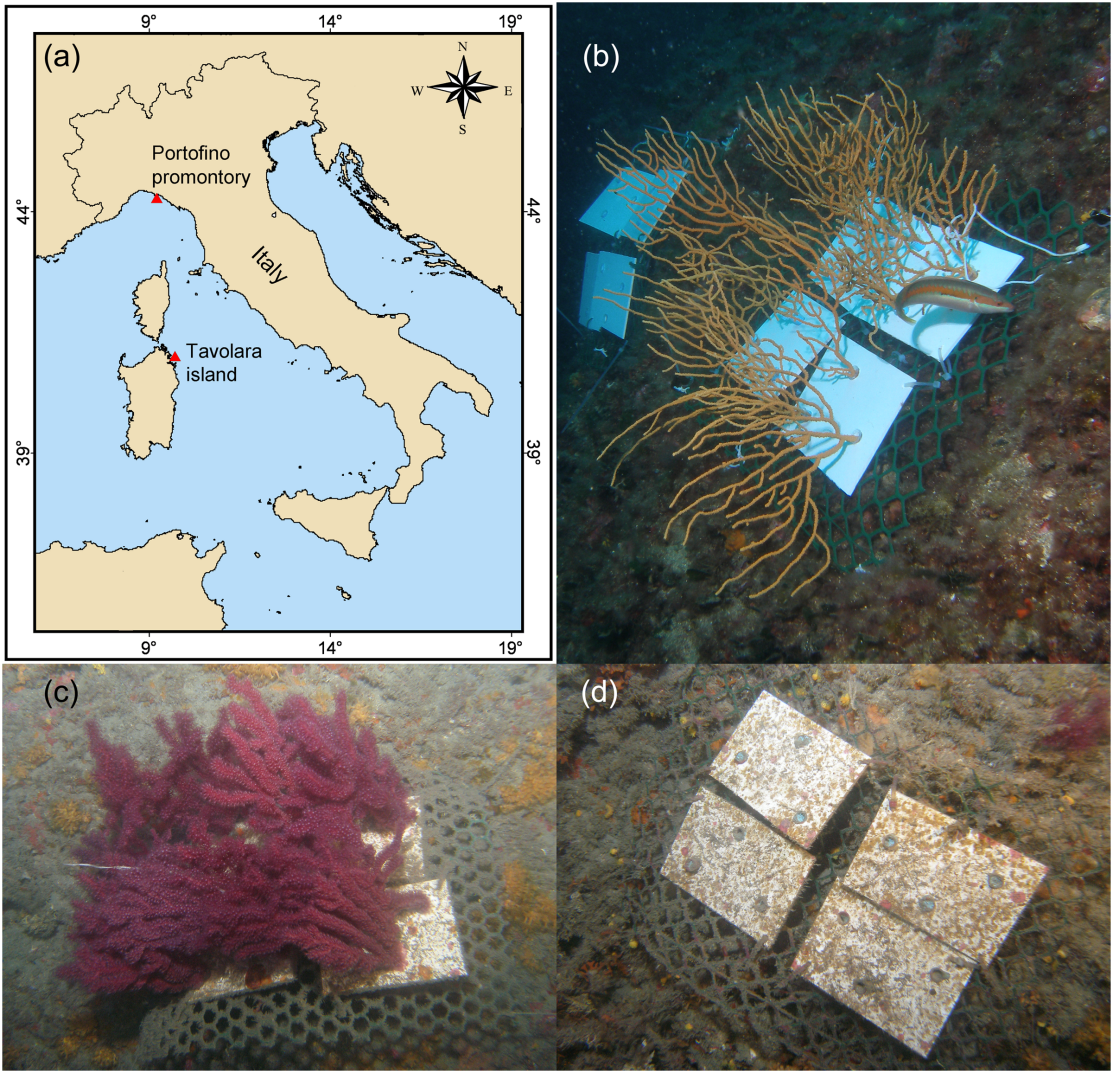
Study sites and experimental set-up. a) Location of the study sites; b) plots with and without (background) *Eunicella cavolinii* at 24 m depth in Portofino at the beginning of the experiment; plots with (c) and without (d) *Paramuricea clavata* at 40 m depth in Portofino at the end of the experiment.

Possible effects of the presence of *Eunicella cavolinii* (Koch, 1887) and *Paramuricea clavata* (Risso, 1826) (Anthozoa, Plexauridae) on the early recruitment of epibenthic organisms were investigated by a manipulative field experiment carried out from June to October, 2010. To limit the impact of the experiments 10.5×15.0 cm foamed PVC (Forex) panels were used to quantify the recruitment. Although artificial panels cannot exactly mimic natural substrates, they are commonly used in experimental ecology to investigate early-stage recruitment and succession processes. This method is simple, inexpensive to implement, and provides results very similar to natural substrates [20], [21], [22], [23]. Gorgonian ‘forests’ were simulated by transplanting three 20 cm long ramified apical branches on each artificial panel. Branches were collected from 40–50 cm high colonies to avoid detaching whole specimens from the nearby populations. The collected apical branches were maintained in continuously renewed seawater without exposing them to air during the transplant operations, which lasted a few hours on the boat. The simulated density of gorgonians was comparable to the maximum values observed in natural patches in terms of abundance (190 colonies m^−2^) for *Eunicella cavolinii* [24] and biomass for *Paramuricea clavata* [6]. Sets of four panels were arranged in plots (Fig. 1b). For *E. cavolinii*, four forested and four non-forested plots were deployed, interspersed, at 24 m depth. Whilst for *P. clavata* the same numbers of plots were installed at 40 m depth. Depths were chosen according to the maximum densities generally occurring in natural populations. Field experiments were simultaneously conducted in two sites with contrasting environmental conditions where dense populations of these gorgonian species naturally occur (Fig. 1a): Tavolara Island (north-western Tyrrhenian Sea, Lat. 40° 54′ 22″ N–Long. 9° 44′ 00″ E) and Portofino Promontory (Ligurian Sea, Lat. 44° 17′ 54″ N–Long. 9° 13′ 09″ E). Tavolara Island is a limestone-dolomite massif with steep cliffs, while Portofino Promontory bedrock is made of a conglomerate of heterogeneous pebbles, mainly of marly limestone, in a sandy-limestone matrix. Compared to Portofino, Tavolara waters are generally more transparent and richer in oxygen, ammonia, total nitrogen and total phosphorus. Portofino waters are richer in nitrates, nitrites and phytoplankton, in terms of Chlorophyll concentration, especially at greater depth (Table 1). To reduce possible anthropogenic disturbances, study sites were selected within Marine Protected Areas.

**Table 1:** Mean values (±s.e., observations number in brackets) of the main water physical and chemical parameters at Tavolara (August 2008–July 2009, from the database of the Italian Ministry of Environment and Protection of Land and Sea, http://www.sidimar.tutelamare.it/) and Portofino (August 2008–August 2009, from the database of the Liguria Region’ Environmental Agency, http://www.ambienteinliguria.it).

		Tavolara		Portofino	
Parameter	Unit	Surface	−24 m	Surface	−24 m
Temperature	°C	18.73±1.38 (12)	17.90±1.10 (12)	19.76±2.07 (7)	17.84±1.27 (7)
Salinity	psu	37.71±0.23 (12)	37.88±0.22 (12)	37.34±0.33 (7)	37.52±0.13 (7)
pH		8.20±0.06 (10)	8.24±0.05 (10)	8.39±0.02 (7)	8.39±0.02 (7)
O_2_	% sat	114.50±3.56 (11)	101.92±3.42 (11)	81.90±0.87 (7)	97.86±1.46 (7)
Chl	µg L^−1^	0.26±0.01 (10)	0.25±0.01 (10)	0.30±0.05 (7)	0.46±0.08 (7)
Transparency	m	19.00±3.43 (12)		11.93±2.01 (7)	
Nitrates	µM L^−1^	0.06±0.02 (5)		0.92±0.49 (7)	
Nitrites	µM L^−1^	0.05±0.04 (5)		0.11±0.11 (7)	
Ammonia	µM L^−1^	0.61±0.21 (5)		0.35±0.22 (7)	
N_tot_	µM L^−1^	12.70±4.08 (5)		7.53±1.18 (7)	
Phosphates	µM L^−1^	0.07±0.04 (5)		0.01±0.01 (7)	
P_tot_	µM L^−1^	0.15±0.08 (5)		0.01±0.01 (7)	
Silicates	µM L^−1^	2.10±0.93 (5)		2.12±0.64 (7)	

Panels were collected after 4 months, before the storms season. Panels were brought to the surface in individual plastic zip-bags and preserved in a buffered solution of formaldehyde (4%) until observation.

### Ethics Statement

Sampling at Tavolara and Portofino was done in accordance with Italian laws, and authorisations were granted by the “Marine Protected Area Tavolara - Punta Coda Cavallo” (www.amptavolara.com) and the “Marine Protected Area of Portofino” (www.portofinoamp.it). This study did not involve endangered or protected species.

### Laboratory and Data Analyses

Sessile species that recruited on the panels were identified to the lowest possible taxonomic level and their percent covers on the upper side was estimated by superimposing a reference grid of 400 equal sized squares [25]. Species richness (as number of species, *S*), species diversity (as Hill’s diversity number *N1* = Exp *H’*, where *H’* is the Shannon’s index based on natural logarithm) and the corresponding evenness component (as *N10* = *N1*/*S*) were calculated for each panel [26]. Differences in assemblage structures were represented using Principal Coordinate Analysis (PCoA unconstrained ordination plot; [27]) based on Bray-Curtis similarities of square root-transformed percent cover data. Subsequently, taxa were pooled into main ecological and taxonomic groups and their contribution to similarity patterns of the assemblages were investigated by multivariate multiple regression using the DistLM procedure [28] and represented by correlation vectors on the PCoA ordination plots.

Differences in community structures, species abundances, ecological and taxonomic group abundances and species diversity indices between forestation treatment (Fo: fixed factor with 2 levels: presence/absence of gorgonian forest), sites (Si: fixed factor with 2 levels: Tavolara/Portofino), and plots (Pl: random factor nested in Fo×Si with 4 levels) were assessed by permutational multivariate analysis of variance (PERMANOVA, α = 0.05; [29]). When less than 999 unique values in the permutation distribution were available, asymptotical Monte Carlo *P*-values was used instead of permutational *P*-values. Multivariate tests were performed on Bray-Curtis similarities of square root-transformed percent cover data, while univariate tests were run on untransformed data using the Euclidean distance. Significant interactions among main factors were investigated by post-hoc pair-wise tests. Statistical analyses were performed using PRIMER 6 with PERMANOVA+ add-on package [30].

## Results

The experiment was monitored periodically and at the end, all transplanted gorgonian branches were alive and in good condition (Fig. 1c, d). Unfortunately, one panel with transplanted branches of *P. clavata* and an entire plot without gorgonian branches associated to the *E. cavolinii* experiment were lost at one site, Portofino. Measurements carried out at the end of the experiment confirmed that the simulated gorgonian density and biomass on the panels were consistent between forested plots and sites. In particular the mean dry mass of *P. clavata* was 827±69 g m^−2^ (±s.e.), which correspond to an ash free dry mass of ∼91 g m^−2^ (according to the conversion factor provided by [31]).

Overall, assemblages recruited on the experimental panels included 233 taxa, of which 146 occurred at 24 m depth, 160 at 40 m depth and 73 were common to both depths. The effect of the two gorgonian species on the recruitment of sessile assemblages was analysed separately.

### Effects of *Eunicella cavolinii*

**Figure 2:**
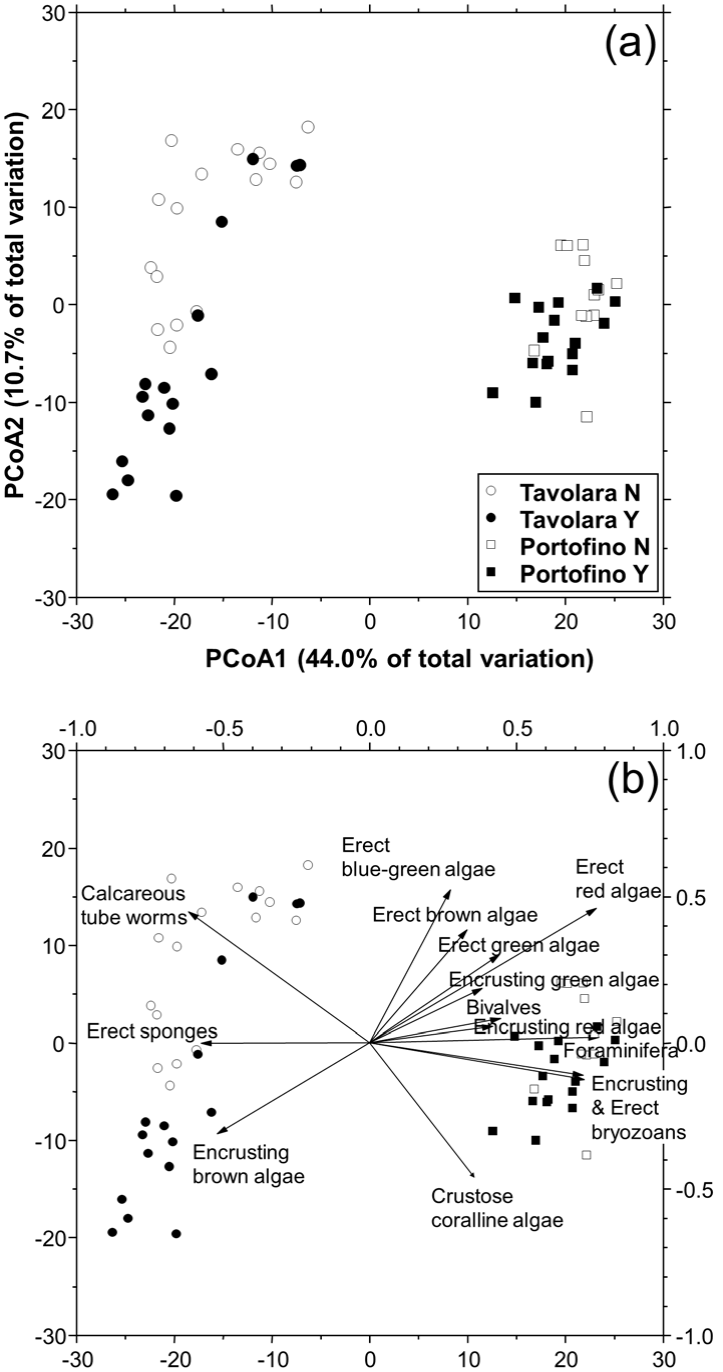
Effects of *Eunicella cavolinii* on benthic assemblages structures. a) PCoA unconstrained ordination plot (i.e. metric MDS) based on Bray-Curtis similarities of square rout-transformed sessile assemblages percent cover on recruitment panels with (Y) and without (N) *Eunicella cavolinii* forest after 4 months in Tavolara and Portofino; b) significant correlations of main ecological groups with PCoA Axes, represented by superimposed vectors (DistLM, marginal test *P<0.01*).

The most abundant taxa on the panels at 24 m depth were encrusting green algae (88.6%±2.0 s.e.), erect red algae (57.6%±4.1 s.e.) and crustose coralline algae (55.4%±1.4 s.e.), followed by foraminifera (29.2%±1.9 s.e.), hydroids (15.3%±1.8 s.e.), erect brown algae (11.0%±1.1 s.e.) and calcareous tube worms (10.2%±0.7 s.e.). Overall, the benthic assemblages differed between plots and sites and were significantly affected by the presence of the gorgonian *E. cavolinii* (Table 2a). The PCoA ordination plot represented 54.7% of the total variation. The PCoA1 axis showed the clear difference between the assemblages at Tavolara and Portofino, while the PCoA2 axis showed the differentiation due to the presence and absence of the gorgonian (Fig. 2a). Although the patterns of differentiation on the PCoA plot are in the same direction, the benthic assemblages at Tavolara appeared more heterogeneous and more affected by the presence of *E. cavolinii* than at Portofino. The correlation vectors, superimposed on the PCoA plot (Fig. 2b), showed the relevance of the ecological and taxonomic groups in the differentiation of the recruited assemblages. As a general trend, erect algae were more abundant on the panels without gorgonians, especially at Portofino, while calcareous tube worms were more abundant on the panels without gorgonians at Tavolara.

**Table 2:** Summary of PERMANOVA test on the effects of (a) *Eunicella cavolinii* and (b) *Paramuricea clavata* forests (Fo) on the recruitment of sessile assemblages at different sites (Si) and among plots (Pl) within Fo×Si (Bray-Curtis similarities on square root transformed data).

Source	*df*	SS	MS	Pseudo-*F*	*P* (perm)	Unique perms
(a) *E. cavolinii*						
Fo	1	2’058	2’058	1.817	0.0347	9’926
Si	1	21’566	21’566	19.045	0.0001	9’914
Fo×Si	1	1601	1’601	1.413	0.1586	9’928
Pl (Fo×Si)	11	12’456	1’132	3.691	0.0001	9’719
Res	45	13’807	307			
Total	59	51’597				
(b) *P. clavata*						
Fo	1	5’345	5’345	3.690	0.0001	9’930
Si	1	33’061	33’061	22.823	0.0001	9’929
Fo×Si	1	4’738	4’738	3.271	0.0003	9’920
Pl (Fo×Si)	12	17’425	1’452	2.754	0.0001	9’703
Res	47	24’779	527			
Total	62	84’386				

**Figure 3:**
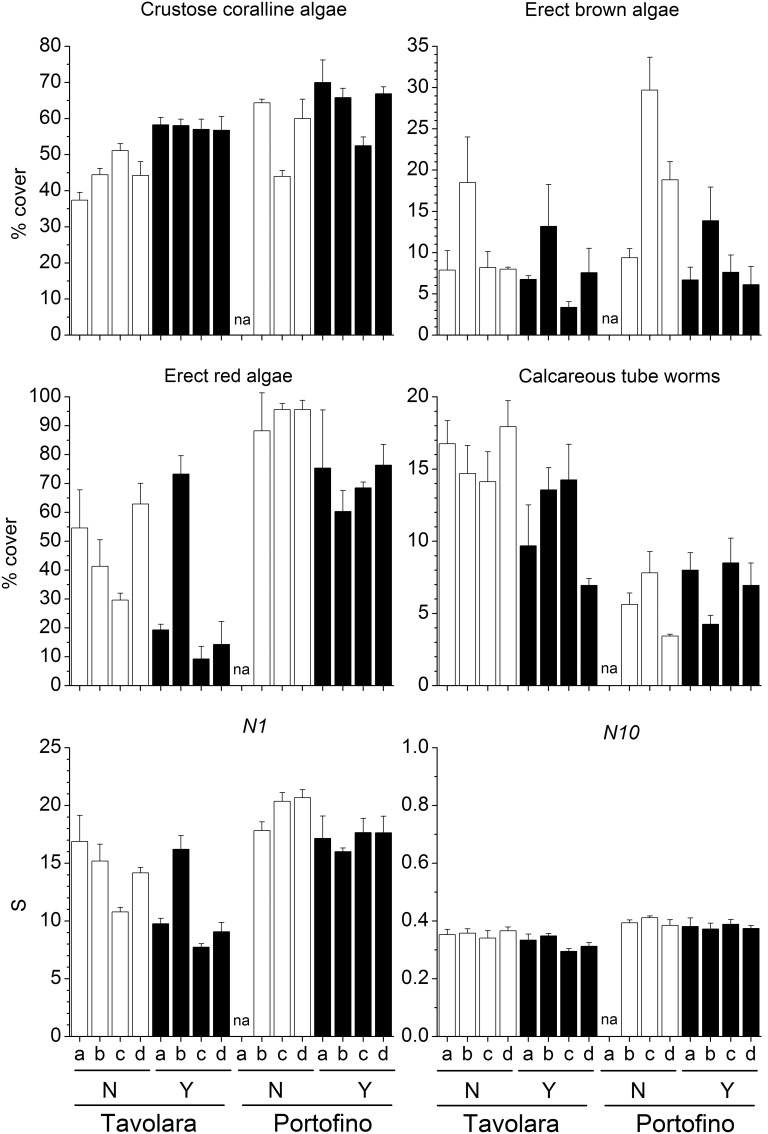
Effects of *Eunicella cavolinii* on selected groups and species diversity indices. Mean (±s.e.) percent cover of selected groups recruited on panels and species diversity indices (Hill’s diversity number *N1* and corresponding evenness component *N10*) for each experimental plot at Tavolara and Portofino, with (Y) and without (N) *Eunicella cavolinii* at 24 m depth (na = plot not available).

**Figure 4:**
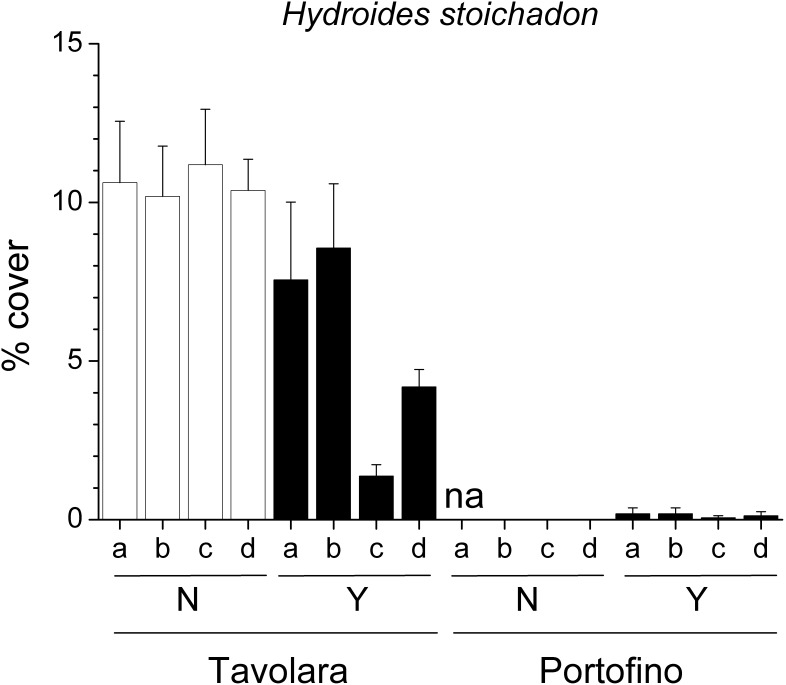
Effects of *Eunicella cavolinii* on the serpulid polychaete *Hydroides stoichadon*. Mean (±s.e.) percent cover of the serpulid polychaete *Hydroides stoichadon* on panels for each experimental plot at Tavolara and Portofino, with (Y) and without (N) *Eunicella cavolinii* at 24 m depth (na = plot not available).

Despite the large local variability, the mean percent cover of several groups was significantly different between the two sites (Table 3). Erect red algae (including 44 taxa, Fig. 3), green algae (12 taxa) and crustose coralline algae (Fig. 3) were significantly more abundant at Portofino, while encrusting brown algae (2 taxa) were more abundant at Tavolara. Among invertebrates, foraminifera (4 taxa), bivalves (i.e. *Anomia ephippium* Linnaeus, 1758), encrusting bryozoans and erect bryozoans (10 and 9 taxa respectively) showed greater percent cover at Portofino. In contrast, erect sponges were more abundant at Tavolara. On panels with *E. cavolinii*, a significant reduction in percent cover of erect red algae (Fig. 3), brown algae (12 taxa, Fig. 3) and green algae was observed, whilst recruitment of crustose coralline algae and erect sponges was enhanced. The mean percent cover of calcareous tube worms (14 taxa, Fig. 3) was significantly reduced by the presence of the gorgonian, but only at one site, Tavolara. Similarly, at the same site, the serpulid polychaete *Hydroides stoichadon* Zibrowius, 1971 was also significantly less abundant in the presence of the gorgonian (pair-wise test: *P*<0.05). However, in contrast at Portofino *H. stoichadon* was occasionally observed but only on the panels which had transplanted gorgonian branches (Fig. 4).

**Table 3:** Summary of PERMANOVA test on the effects of *Eunicella cavolinii* forests (Fo) on the recruitment of ecological and functional groups and diversity indices at different sites (Si) and among plots (Pl) within Fo×Si.

	Forest (Fo)			Site (Si)			Fo×Si			Plot (Fo×Si)			Res
	(1 *df*)			(1 *df*)			(1 *df*)			(11 *df*)			(45 *df*)
	MS	*F*	*P*	MS	*F*	*P*	MS	*F*	*P*	MS	*F*	*P*	MS
Erect blue-green algae	377.74	3.25	0.0949 ns	65.39	0.56	0.4905 ns	57.55	0.50	0.5193 ns	116.14	3.65	0.0006***	31.81
Erect red algae	6’238.30	4.89	0.0337*	28’074.00	22.03	0.0001***	90.40	0.07	0.8214 ns	1’274.50	4.19	0.0005***	304.05
Encrusting red algae	0.12	0.06	0.8071 ns	9.14	4.93	0.0510 ns	0.02	0.01	0.9251 ns	1.85	2.55	0.0096**	0.73
Crustose coralline algae	1’611.40	8.76	0.0133*	1’210.90	6.58	0.0266*	113.60	0.62	0.4505 ns	183.99	4.83	0.0002***	38.07
Erect brown algae	686.49	5.00	0.0475*	333.96	2.43	0.1419 ns	224.16	1.63	0.2367 ns	137.22	4.11	0.0002***	33.35
Encrusting brown algae	7.46	0.78	0.4479 ns	50.63	5.29	0.0105*	7.80	0.81	0.4364 ns	9.58	9.95	0.0001***	0.96
Encrusting green algae	1’069.00	2.42	0.1427 ns	2’025.50	4.58	0.0567 ns	16.43	0.04	0.8566 ns	442.65	2.84	0.0062**	155.64
Erect green algae	7.36	11.22	0.0057**	5.82	8.88	0.0117*	0.00	0.01	0.9317 ns	0.66	1.64	0.1153 ns	0.40
Foraminifera	703.38	3.12	0.1119 ns	7’915.90	35.06	0.0001***	406.27	1.80	0.2165 ns	225.79	4.85	0.0001***	46.60
Erect sponges	11’245.00	3.85	0.0056**	42’098.00	14.42	0.0001***	1’358.40	0.47	0.8051 ns	2’919.30	3.82	0.0001***	763.53
Hydroids	462.56	1.07	0.3229 ns	1’002.10	2.33	0.1493 ns	135.47	0.31	0.5824 ns	430.45	3.85	0.0006***	111.72
Calcareous tube worms	44.43	1.87	0.2001 ns	769.63	32.48	0.0003***	135.71	5.73	0.0358*	23.70	2.19	0.0315*	10.80
Bivalves	0.14	3.07	0.1112 ns	0.63	14.01	0.0002***	0.14	3.07	0.1008 ns	0.04	1.06	0.4171 ns	0.04
Encrusting bryozoans	10.13	1.89	0.2187 ns	234.47	43.70	0.0001***	5.48	1.02	0.3367 ns	5.37	1.78	0.0649 ns	3.01
Stoloniferous bryozoans	0.67	0.01	0.9110 ns	0.89	0.02	0.9010 ns	128.35	2.68	0.1343 ns	47.86	2.26	0.0183*	21.16
Erect bryozoans	4.09	0.36	0.5626 ns	316.64	27.66	0.0002***	15.08	1.32	0.2861 ns	11.45	4.28	0.0002***	2.67
*S*	513.39	3.34	0.0923 ns	1’773.60	11.54	0.0029**	27.08	0.18	0.6911 ns	153.66	6.43	0.0001***	23.90
*N1*	135.93	5.40	0.0370*	512.72	20.38	0.0003***	4.10	0.16	0.7033 ns	25.16	4.98	0.0001***	5.05
*N10*	0.01	9.91	0.0087**	0.04	39.23	0.0001***	0.00	0.81	0.3903 ns	0.00	0.79	0.6509 ns	0.00

Overall, species richness (*S*) was significantly higher at Portofino, but was not affected by the presence of the *E. cavolinii* (Table 3). Furthermore, species heterogeneity (*N1*) and evenness (*N10*) were on average higher at Portofino, and were significantly higher in panels that did not have gorgonians present (Fig. 3).

### Effects of *Paramuricea clavata*

**Figure 5:**
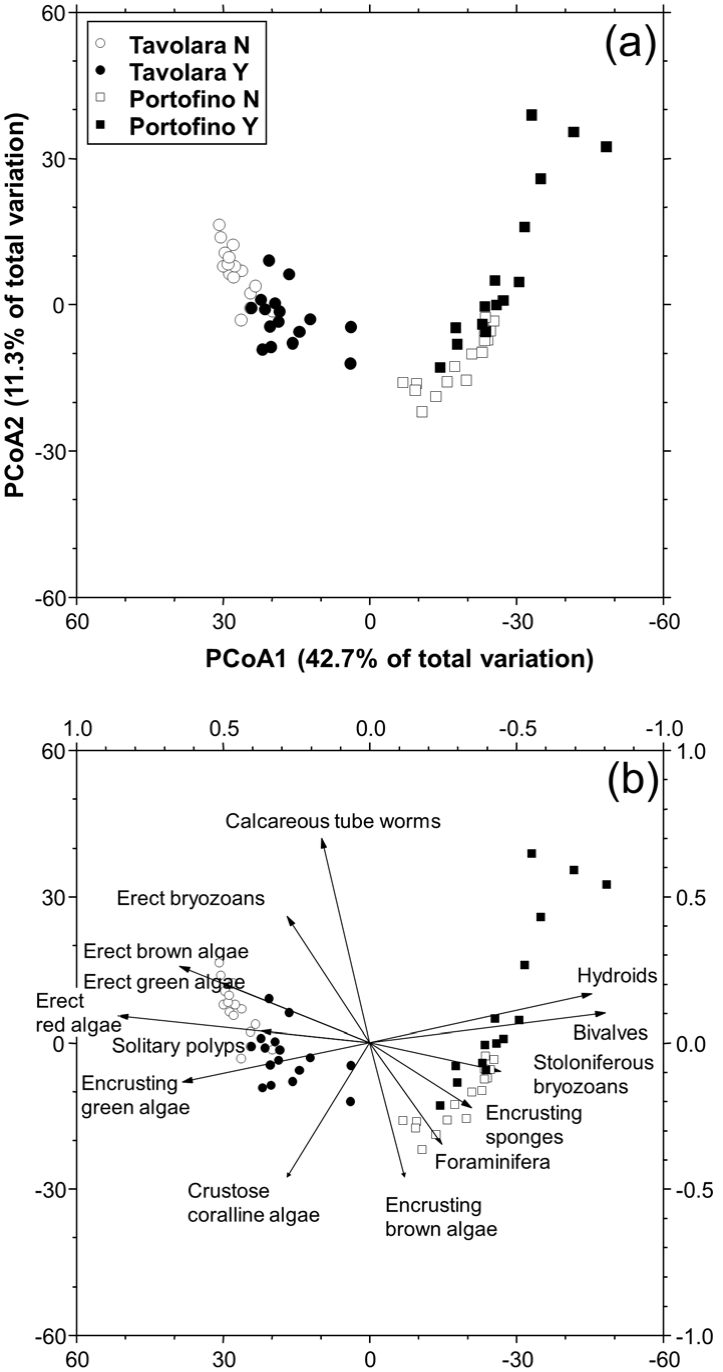
Effects of *Paramuricea clavata* on benthic assemblages structures. a) PCoA unconstrained ordination plot (i.e. metric MDS) based on Bray-Curtis similarities of square rout-transformed sessile assemblages percent cover on recruitment panels with (Y) and without (N) *Paramuricea clavata* forest after 4 months in Tavolara and Portofino; b) significant correlations of main ecological groups with PCoA Axes, represented by superimposed vectors (DistLM, marginal test *P<0.01*).

The most abundant taxa on the panels at 40 m depth were encrusting brown algae (59.8%±3.8 s.e.) and small erect red algae (56.7%±5.2 s.e.), followed by encrusting green algae (28.6%±2.3 s.e.), foraminifera (26.5%±1.4 s.e.), crustose coralline algae (23.5%±1.8 s.e.) and hydroids (20.2%±3.3 s.e.). The recruited assemblages differed notably between plots and between sites and the assemblages were significantly affected by the combination of presence/absence of the gorgonian *P. clavata* and site (Table 2b). The PCoA plot (which displays 54.0% of the total variation) shows both the difference between Tavolara and Portofino, mainly along the PCoA1 axis, and a clear shift in the assemblage structures with relation to the presence of the gorgonian forest (Fig. 5a).

The correlation vectors, superimposed on the PCoA plot (Fig. 5b), show the relevance of the ecological and taxonomic groups in the differentiation of the recruited assemblages. At Tavolara, the groups of erect algae were more abundant on the panels without gorgonians, and crustose coralline algae tended to increase on forested panels. At Portofino, foraminifera and encrusting brown algae were more abundant on non- forested panels, while hydroids and bivalves were found in greater numbers on the forested panels.

**Figure 6:**
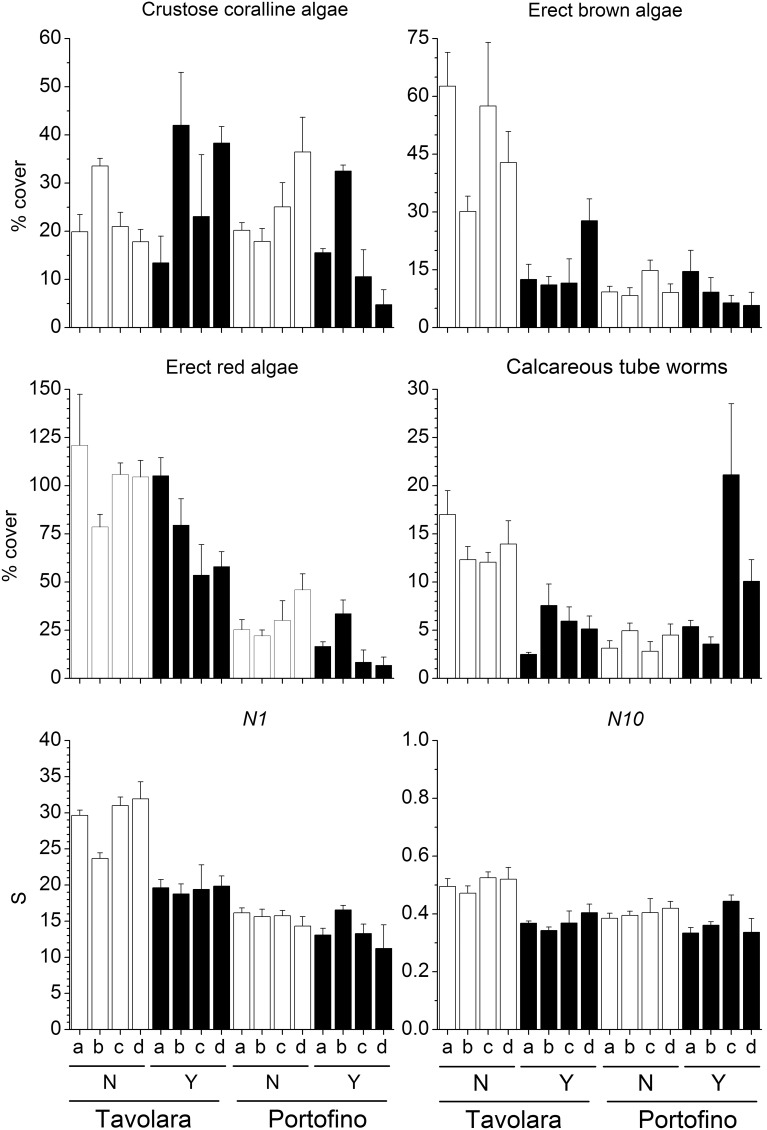
Effects of *Paramuricea clavata* on selected groups and species diversity indices. Mean (±s.e.) percent cover of selected groups recruited on panels and species diversity indices (Hill’s diversity number *N1* and corresponding evenness component *N10*) for each experimental plot at Tavolara and Portofino, with (Y) and without (N) *Paramuricea clavata* at 40 m depth.

**Figure 7:**
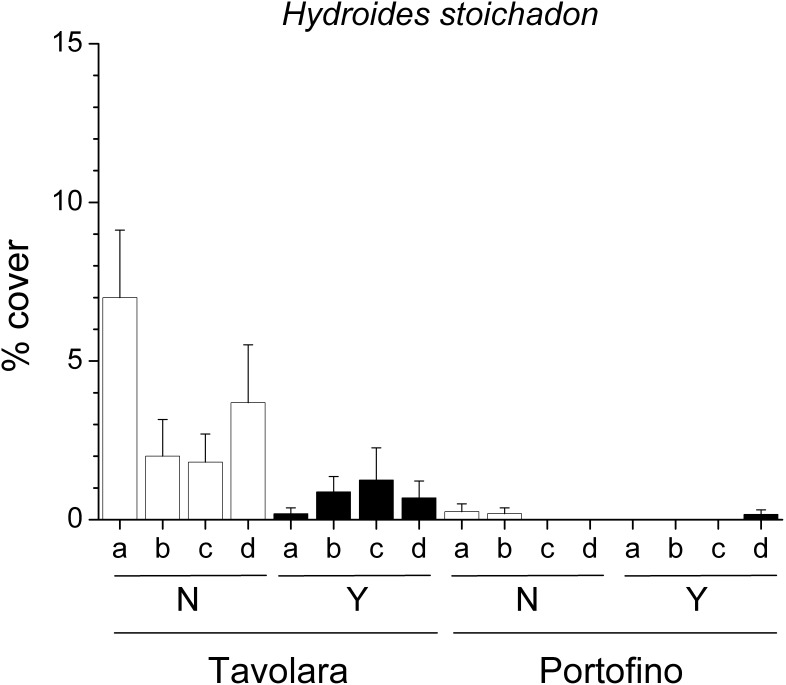
Effects of *Paramuricea clavata* on the serpulid polychaete *Hydroides stoichadon*. Mean (±s.e.) percent cover of the serpulid polychaete *Hydroides stoichadon* on panels for each experimental plot at Tavolara and Portofino, with (Y) and without (N) *Paramuricea clavata* at 40 m depth.

Total percent cover of most groups showed large variability between plots, but also showed some site specific effects related to presence of gorgonians (Table 4). Erect red algae (including 38 taxa) were more abundant in Tavolara than in Portofino and were significantly lowered by the presence of gorgonian (Fig. 6). In contrast, foraminifera (3 taxa) were more abundant in Portofino but were also reduced on the forested panels. Encrusting green algae (6 taxa) were more abundant at Tavolara, while crustose coralline algae did not show any effects of site or gorgonians presence (Fig. 6). Overall, solitary polyps were found only at Tavolara, while hydroids (12 taxa), bivalves (i.e. *Anomia ephippium* Linnaeus, 1758) and stoloniferous bryozoans (2 taxa) were much more abundant at Portofino. Encrusting bryozoans (7 taxa) were facilitated by gorgonians at both sites. Even with different mean percent covers, erect brown (15 taxa, Fig. 6) and green algae (10 taxa), along with calcareous tube worms (18 taxa), were significantly more abundant on panels without gorgonians at Tavolara (pair-wise test: *P*<0.01), whilst these effects were not observed at Portofino. Conversely encrusting brown algae (5 taxa) were facilitated by the gorgonian at Tavolara (pair-wise test: *P*<0.05), and reduced at Portofino (pair-wise test: *P*<0.05). Encrusting sponges (3 taxa) were absent at Tavolara, yet were significantly more abundant on the panels without gorgonian at Portofino (pair-wise test: *P*<0.05). As observed in the experiment with *E. cavolinii*, at 24 m depth, even if less abundant at 40 m depth, the serpulid polychaete *Hydroides stoichadon* was significantly reduced by the gorgonian at Tavolara (pair-wise test: *P*<0.05) (Fig. 7).

**Table 4:** Summary of PERMANOVA test on the effects of *Paramuricea clavata* forests (Fo) on the recruitment of ecological and functional groups and diversity indices at different sites (Si) and among plots (Pl) within Fo×Si.

	Forest (Fo)			Site (Si)			Fo×Si			Plot (Fo×Si)			Res
	(1 *df*)			(1 *df*)			(1 *df*)			(12 *df*)			(47 *df*)
	MS	*F*	*P*	MS	*F*	*P*	MS	*F*	*P*	MS	*F*	*P*	MS
Erect red algae	7’211.80	6.45	0.0311*	65’548.00	58.60	0.0002***	761.73	0.68	0.4255 ns	1’121.20	2.51	0.0107*	446.55
Encrusting red algae	245.90	0.78	0.3867 ns	138.87	0.44	0.5185 ns	1’093.20	3.49	0.0845 ns	314.50	3.47	0.0015**	90.68
Crustose coralline algae	33.10	0.08	0.7776 ns	521.02	1.22	0.2757 ns	903.81	2.11	0.1727 ns	428.53	3.46	0.0009***	123.75
Erect brown algae	4’513.00	14.80	0.0030**	7’803.00	25.59	0.0006***	3’803.60	12.48	0.0064**	305.48	2.01	0.0443*	152.10
Encrusting brown algae	2’006.70	1.55	0.2372 ns	6’170.20	4.75	0.0553 ns	17’609.00	13.56	0.0025**	1’302.10	4.11	0.0001***	316.47
Erect green algae	117.86	17.48	0.0020**	106.08	15.74	0.0027**	76.62	11.37	0.0078**	6.75	1.37	0.2077 ns	4.94
Encrusting green algae	2’070.00	4.48	0.0591 ns	6’343.10	13.74	0.0024**	33.45	0.07	0.7881 ns	462.91	3.03	0.0027**	152.79
Foraminifera	1’317.80	23.27	0.0010**	1’023.40	18.07	0.0026**	0.84	0.01	0.9076 ns	56.53	0.65	0.7833 ns	86.61
Encrusting sponges	0.11	12.33	0.0055**	0.44	49.32	0.0003***	0.11	12.33	0.0057**	0.01	0.18	0.9993 ns	0.05
Solitary polyps	0.01	0.62	0.4943 ns	0.12	8.28	0.0114*	0.01	0.62	0.4918 ns	0.01	1.28	0.2409 ns	0.01
Hydroids	1’305.10	1.27	0.2843 ns	20’172.00	19.61	0.0011**	757.75	0.74	0.4075 ns	1’032.10	5.39	0.0002***	191.32
Calcareous tube worms	21.72	0.30	0.6199 ns	107.14	1.47	0.2578 ns	851.29	11.71	0.0018**	72.90	3.31	0.0015**	22.04
Bivalves	49.73	1.52	0.2550 ns	1’256.40	38.30	0.0001***	72.87	2.22	0.1639 ns	32.90	4.48	0.0001***	7.35
Encrusting bryozoans	184.08	8.60	0.0121*	44.36	2.07	0.1715 ns	1.91	0.09	0.7676 ns	21.45	2.60	0.0094**	8.24
Stoloniferous bryozoans	88.99	1.74	0.2201 ns	459.42	9.00	0.0079**	116.52	2.28	0.1627 ns	51.12	1.95	0.0473*	26.26
Erect bryozoans	55.41	0.32	0.5747 ns	608.17	3.47	0.0898 ns	63.02	0.36	0.5509 ns	175.83	3.86	0.0009***	45.50
*S*	253.39	2.78	0.1171 ns	4’474.30	49.00	0.0003***	40.33	0.44	0.5259 ns	91.50	2.27	0.0198*	40.23
*N1*	524.67	27.65	0.0004***	1’487.80	78.41	0.0003***	233.77	12.32	0.0054**	19.01	1.92	0.0562 ns	9.88
*N10*	0.11	26.09	0.0005***	0.04	10.50	0.0112*	0.04	9.72	0.0062**	0.00	1.31	0.2454 ns	0.00

Overall, species richness (*S*) was significantly higher at Tavolara, but it was not affected by the presence of the gorgonian (Table 4). Furthermore, species heterogeneity (*N1*) and evenness (*N10*) were significantly higher in absence of the gorgonian at Tavolara (pair-wise test: *P*<0.01) whilst no effects were found at Portofino (Fig. 6).

## Discussion

Owing to the use of experimental panels, in 4 months of deployment it was possible to detect the effects of the two most common Mediterranean gorgonians on the early-stage recruitment of the epibenthic assemblages. The newly recruited assemblages differed amongst plots and between sites, both in *Eunicella cavolinii* (24 m depth) and in *Paramuricea clavata* (40 m depth) treatments. Analyses of the recruitment highlighted site-specific and species-specific effects of the gorgonian forests. Overall, the presence of gorgonians limited the growth of erect algae and, at Tavolara, the recruitment of serpulid polychaetes. Moreover, the presence of gorgonians, lowered species diversity and evenness of the assemblages on the experimental panels. These effects may be related to both biological and physical effects. Being passive filter feeders (they cannot create currents on their own [32]) and active predators (polyps capture preys by nematocysts) the gorgonians can intercept and feed on drifting larvae before settlement. Coma et al. [33] documented that *P. clavata* has a broad and heterogeneous diet with prey species ranging from 3.8 µm (nanoeukaryotes) to 700 µm (copepods). Protozoans and microalgae are the main items caught by the polyps. Capture of detrital and live particulate organic carbon is minimal in the summer, due to the scarcity of suspended food during this season [34]. Gorgonians may also compete with other filter-feeders for food and/or for space, producing allelochemicals [35]. On the other hand, gorgonians could affect settlement and recruitment processes by modifying microscale hydrodynamic conditions, sediment deposition rates by limiting resuspension processes, and algal development by creating shading effects that may reduce the availability of light. In the mesophotic zone, gorgonian forests are considered to be of paramount importance to support surrounding species diversity. It was observed that they significantly increased the deposition of bioavailable substrates and enhanced benthic diversity, compared to areas without colonies [5]. As a result, organisms living in habitats characterised by the presence of these ‘ecosystem engineers’ (*sensu* [36]) may experience a sort of buffer zone where environmental modifications occur more slowly and within narrower ranges with respect to the surrounding ‘unforested’ environment.

In the present study, significant spatial variation of recruitment patterns was observed between Tavolara and Portofino. These differences could be related to variability in larval supply, chemical-physical parameters, sediment deposition and hydrodynamic conditions between sites. For example, in Portofino the value of nitrites and nitrates is higher compared to Tavolara. These differences are likely related to the fact that Tavolara is further from rivers and densely populated areas. Furthermore, at the specific study site (i.e. Punta del Faro) the Portofino Promontory receives a secondary branch of the main Ligurian current [17], transporting urban waste-waters from the Tigullio Gulf, especially during the tourist summer season.

Longer experiments are needed to better understand the temporal dynamics of coralligenous assemblages; nevertheless, this short-time experiment has provided clear indications on the effect of gorgonians on the recruitment trajectories. In the study sites, the presence of gorgonians mainly reduces the initial growth of erect algae in favour of encrusting ones, which are the most important builders of the coralligenous frameworks [1], and some sessile invertebrates. Therefore, depending on the trophic conditions and turbidity of the water, rarefaction of gorgonians may favour a simplification of the coralligenous assemblages and a shift toward more autotrophic communities, dominated by fast growing filamentous algae instead of the slow accretion of crustose coralline algae.

Coralligenous habitats are threatened by several global stressors. The most evident, are climate change (massive mortalities related to temperature anomalies) and fishing activities (mechanical injuries and sediment re-suspension). Recently potential synergism between these stressors have been hypothesized [12], resulting in a fragmentation of the habitat that can open new space available for invasive species. Unfortunately, the dearth of information available on the interactions between octocorals and associated fauna prevents predicting any effect of gorgonian loss on the trophic structure. Owing to natural or anthropogenic causes, the loss of the 3D structure of the epibenthic layer in coralligenous assemblages [3], may affect both the established assemblages and the settlement and recruitment processes. The main question addressed by this study is whether the rapid loss of complexity due to extensive Mediterranean gorgonian mortalities [37], can affect the early-stage recruitment of coralligenous assemblages. Although the biological interaction between gorgonians and the other species deserve further studies, modifications of the edaphic conditions caused by the gorgonians forests influences larval settlement and recruitment processes of the benthic assemblages.

Coralligenous habitats are mainly built by deposition of calcareous thalli of crustose coralline algae, a process that has lasted for thousands of years [1]. These assemblages are generally characterised by high spatial heterogeneity and limited temporal variability, possibly related to the longevity and slow growth rates of the most abundant and structuring species [38], [39], [40], [41]. If the local disappearance of gorgonians causes a shift of the epibenthic assemblages from crustose coralline algae to filamentous algae dominated, complexity decreases. In absence of gorgonians, the resilience of coralligenous bioconstructions could be compromised due to the reduced recruitment of the main builder organisms and other key species. As another possible consequence, the establishment of non-indigenous species, such as the erect green algae *Caulerpa cylindracea* Sonder and the turf forming red algae *Womersleyella setacea* (Hollenberg) R.E.Norris, may be facilitated [42], [43], irremediably altering these unique Mediterranean underwater landscapes. A regional scale assessment of coralligenous habitats taking in account not only the biological component but also the three-dimensional arrangement [44] is needed to develop adequate management plans.
